# Revealing new insights into different phosphorus-starving responses between two maize (*Zea mays*) inbred lines by transcriptomic and proteomic studies

**DOI:** 10.1038/srep44294

**Published:** 2017-03-09

**Authors:** Huimin Jiang, Jianfeng Zhang, Zhuo Han, Juncheng Yang, Cailin Ge, Qingyu Wu

**Affiliations:** 1 Institute of Agricultural Resources and Regional Planning, the Chinese Academy of Agricultural Sciences, Beijing 100081, People’s Republic of China; 2 College of Bioscience and Biotechnology, Yangzhou University, Yangzhou 225009, People’s Republic of China

## Abstract

Phosphorus (P) is an essential plant nutrient, and deficiency of P is one of the most important factors restricting maize yield. Therefore, it is necessary to develop a more efficient program of P fertilization and breeding crop varieties with enhanced Pi uptake and use efficiency, which required understanding how plants respond to Pi starvation. To understand how maize plants adapt to P-deficiency stress, we screened 116 inbred lines in the field and identified two lines, DSY2 and DSY79 that were extreme low-P resistant and sensitive, respectively. We further conducted physiological, transcriptomic, and proteomic studies using the roots of DSY2 and DSY79 under normal or low-P conditions. The results showed that the low-P resistant line, DSY2 had larger root length, surface area and volume, higher root vitality, as well as acid phosphatase activity as compared with the low-P sensitive line, DSY79 under the low-P condition. The transcriptomic and proteomic results suggest that dramatic more genes were induced in DSY2, including the plant hormone signaling, acid phosphatase, and metabolite genes, as compared with DSY79 after being challenged by low-P stress. The new insights generated in this study will be useful toward the improvement of P-utilize efficiency in maize.

Phosphorus (P) is an essential plant nutrient required for all cellular metabolic processes during plant development, reproduction, and environmental adaptation^1^. P is a key structural component of many biological molecules, including nucleic acids, phospholipids, ATP and NADPH^2^. Additionally, P plays a crucial role in controlling the signal transduction cascades participating in the phosphorylation and de-phosphorylation cycles of signaling proteins^3^. P is also involved in physiological events such as photosynthesis, oxidative respiration, carbon and nitrogen assimilation, energy transfer, metabolic regulation, and protein activation^3^. Therefore, P is indispensable for crop production and limited P causing growth yield penalty, and P-deprived crops show reduced growth due to defects in cell division and elongation, a reduction in size and numbers of flowers and seeds, decreasing to a great extent crop productivity^3^.

Despite the importance of P to crops, the concentrations of available inorganic phosphate (Pi) in soil solution are typically very low, due to its propensity to bind strongly to soil surfaces or form insoluble complexes with cation^4^. Thus, deficiency of P is a major constraint for plant growth and crop productivity^5^. To sustain high productivity levels, a continuous Pi input in the form of fertilizers is required. However, recovery of Pi fertilizer by crops is very low because more than 80% becomes immobile and unavailable for plant uptake^1^. Thus, excessive Pi leaches into groundwater, polluting the environment, destroying the soil, causing severe environmental problems. Therefore, to develop a more sustainable agricultural system, it is necessary to develop a more efficient program of P fertilization and breeding crop varieties with enhanced Pi uptake and use efficiency, which required understanding how plants respond to Pi starvation.

Some plants have evolved sophisticated strategies to adapt to P deficiency, including modification of root architecture, secreting organic acids, improving P recycling, and turning on a number of genes involved in low-P adaptation^5^,^6^. However, the complete signaling pathways, as well as the putative phosphate receptors, remain elusive. Works on the model plant *Arabidopsis thaliana* have been very successful in determining the sequence of morphological, physiological and molecular processes underlying low-P adaption; however, different regulatory components may be present in the monocot crops, such as maize, to cope with P starving. Therefore, it is necessary to understand the mechanisms controlling P use efficiency (PUE) in the pursuit of improved crop productivity and sustained agriculture.

To gain more insights into how maize plants adapt to low-P stress, we conducted physiological, transcriptomic and proteomic studies using two contrasting maize inbred lines that showed extremely tolerant or sensitive to P-deficiency from our field screen. The low P-tolerant inbred line DSY2 displayed higher root biomass and root activity as compared with the low-P sensitive line DSY79. The transcriptomic and proteomics studies identified the differentially expressed genes and proteins between the two inbred lines under normal or low-P conditions. The new insights generated from maize transcriptome and proteome in response to P-starvation would be useful toward the improvement of P utilize efficiency in maize.

## Results

### Identification of low-P tolerant and sensitive inbred lines

**Figure 1:**
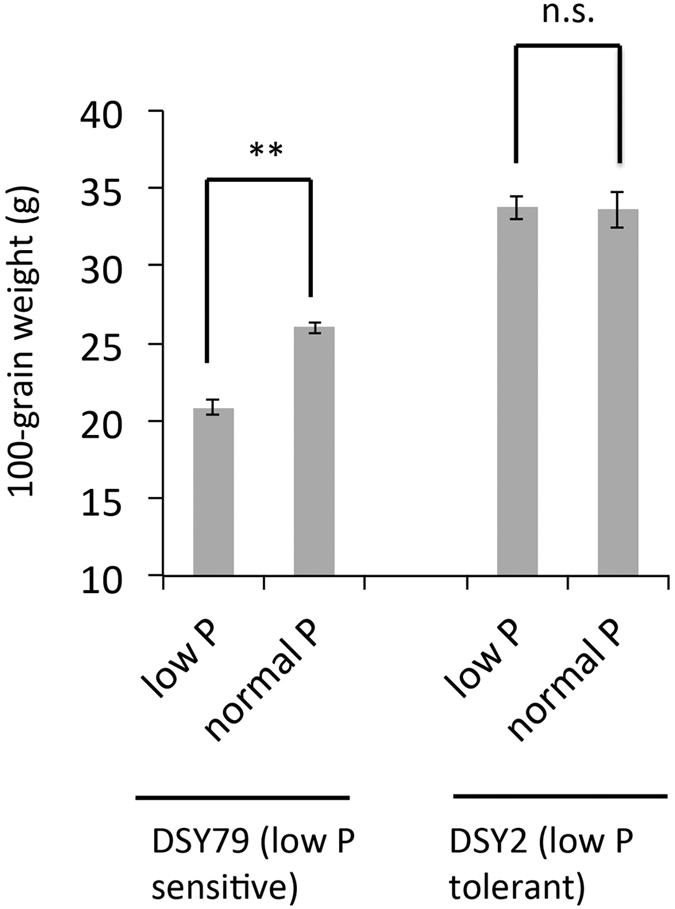
The 100-grain weight of the low-P tolerant line DSY2 and low-P sensitive line DSY79 under normal or low-P conditions. For normal P treatment, 120 kg ha^−1^ P_2_O_5_ was applied, whereas no P_2_O_5_ was applied for the low-P treatment. The data represent the means ± S.D. (standard deviation) for three biological replicates. **p < 0.01 for significant differences according to two-tailed Student’s *t*-test.

To identify the low-P tolerant inbred lines, we previously screened the 116 inbred lines in the field with normal P or low P conditions^7^. The soil used for the experiment contains relatively low P with total P content as 0.69 g/kg and the available P content as 2.77 mg/kg. We applied 120 kg ha^−1^ P_2_O_5_ as normal P treatment, whereas no P_2_O_5_ was applied for the low-P treatment. We identified DSY2 and DSY79 as two extreme low-P tolerant and low-P sensitive lines, respectively, based on the 100-grain weight, an indicator of low-P tolerance. The results suggest that the 100-grain weight of DSY-79 was significantly reduced under the low-P condition as compared with the normal-P condition, whereas the that of DSY-2 was not much affected by the low-P stress, indicating the DSY2 inbred line is more tolerant to low-P stress as compared with DSY79 (Fig. 1). Therefore, we focused on DSY-2 and DSY-79 to studied the mechanisms underlining their different low-P responsiveness.

### The root characters of DSY2 contribute to its low-P tolerance

**Figure 2:**
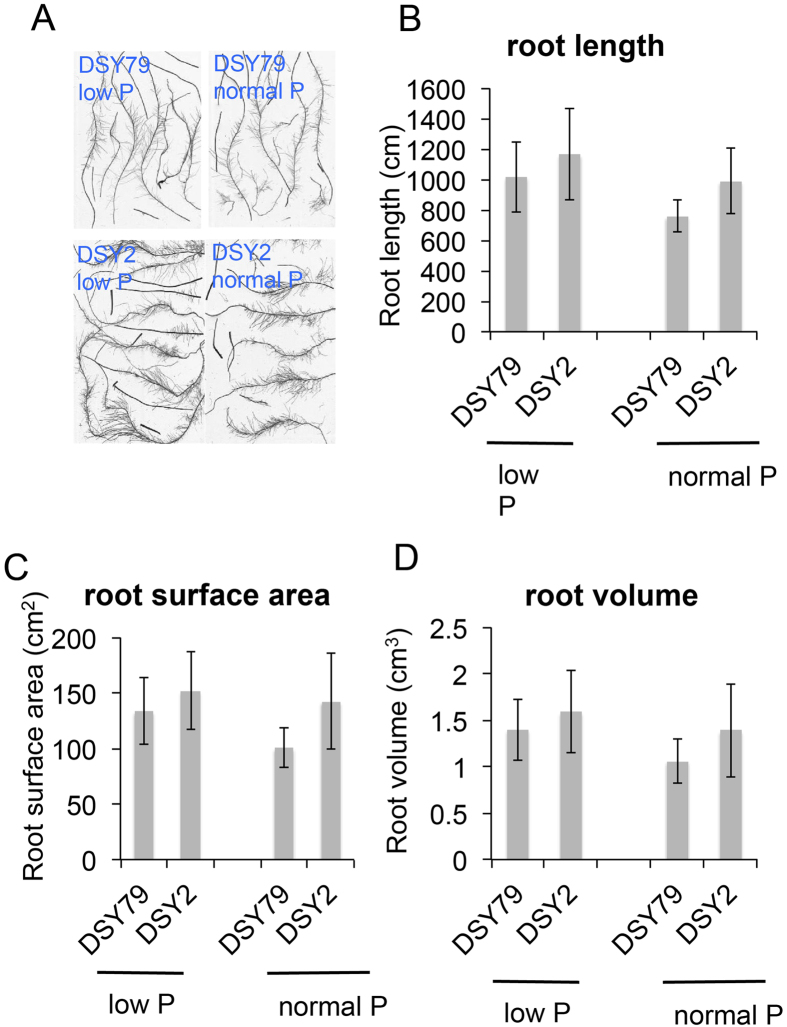
The root morphology of DSY2 and DSY79 under normal or low-P conditions. (**A**) Representative images acquired from the DSY2 and DSY79 roots under normal or low-P conditions. Root length (**B**), root surface area (**C**), and root volume (**D**) of DSY2 and DSY79 under normal or low-P conditions. The 3-leaf-age maize seedlings were cultured in Hoagland’s solution with or without P for 10 d, then the roots were scanned and images were analyzed. The data represent the means ± S.D. (standard deviation) for three biological replicates.

**Figure 3:**
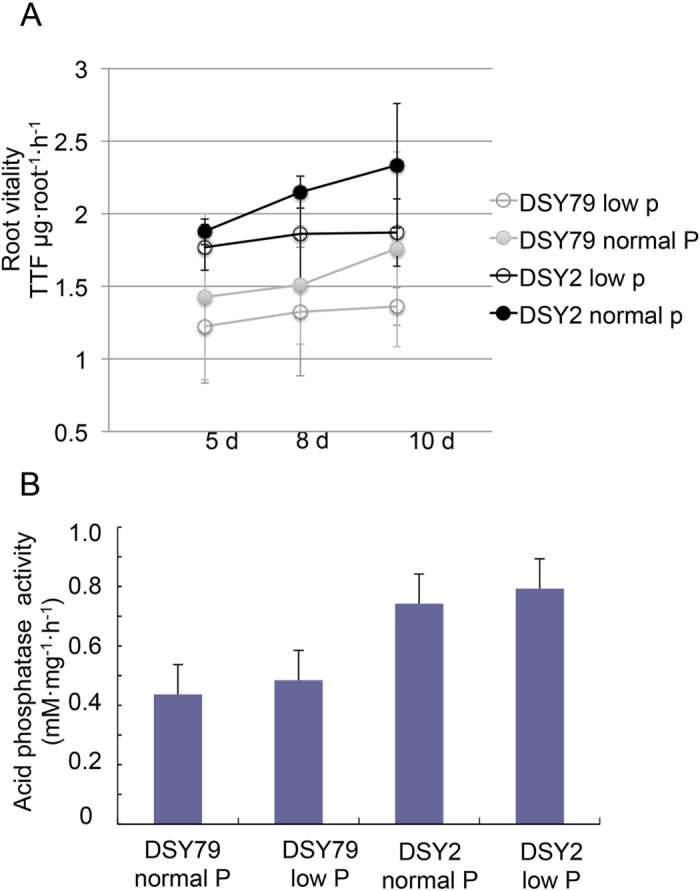
The root vitality and acid phosphatase activity of DSY2 and DSY79 under normal or low-P conditions. (**A**) The root vitality of DSY2 and DSY79 after being cultured in Hoagland’s solution with or without P for 5, 8, or 10 d. (**B**) The acid phosphatase activity of after being cultured in Hoagland’s solution with or without P for 10 d. The data represent the means ± S.D. (standard deviation) for three biological replicates.

The plasticity of root architecture in response to P is a crucial component of plant’s P foraging capacity^2^. One of the strategies for plants responds to low-P stress is altering the root morphology^5^. Thus, we measured the root characters of DSY2 and DSY79 under both low- and normal- P conditions to study how their roots responded to low-P stress. The results showed that the low-P stress enhanced the total root length, surface area and volume of both DSY2 and DSY79 (Fig. 2). However, the low-P tolerant line DSY2 displayed longer root length, larger root surface area, and root volume than DSY79 under both normal and low-P conditions (Fig. 2), suggesting the low-P tolerance of DSY2 is partially attribute to its robust root system, leading to higher accessibility to P. We further asked if the DSY2 and DSY79 had different root vitality under normal and low-P conditions. We measured the root vitality of both lines at 5, 8, and 10 days after being treated by low or normal-P. The results showed that both lines maintained higher root vitality in the normal-P than the low-P condition, and DSY-2 exhibited significantly higher root vitality as compared with DSY79 under both conditions (Fig. 3A).

To provide enough phosphate (Pi) to maintain the metabolism under P-deficient conditions, one of the biochemical strategies that plants take is an increase of Pi mobilization and recycling activity within the plants, such as hydrolysis of phospholipids by acid phosphatases^5^. Therefore, we measured the acid phosphatase activity of both lines under either normal of low-P conditions. The results showed that the acid phosphatase activity of DSY2 was significantly higher that that of DSY79 (Fig. 3B). All the above data suggest that the morphological and physiological characters of DSY2 roots contribute to the low-P tolerance.

### Gene expression profiles of DSY2 and DSY79 under normal and low-P conditions

**Figure 4:**
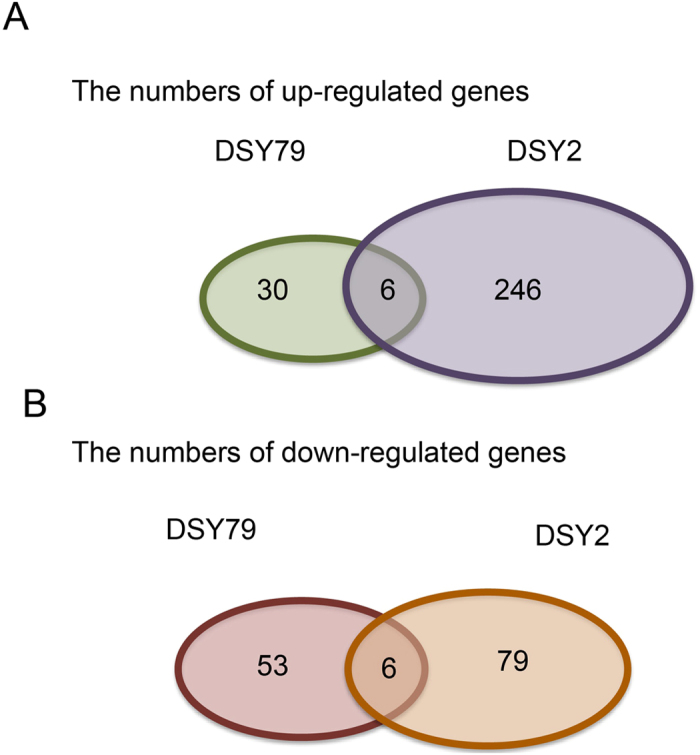
The number of differentially expressed genes of DSY2 and DSY79 under low-P as compared with normal-P conditions.

To gain more insights into the mechanisms of low-P responses of the two inbred lines, we compared the root gene expression profiles under normal or low-P conditions. We focused on the fold change larger than two for further analysis. The transcriptomic results showed that there were only 36 genes induced more than 2-folds in the low-P sensitive line DSY79, whereas 252 genes were induced in the low-P tolerant line DSY2 by the low-P stress (Fig. 4A). Surprisingly, there were only 6 overlapped genes induced in both inbred lines by the low-P stress, suggesting their responsive mechanisms are very different. Moreover, the results indicate that DSY2 was more responsive to the low-P stress. The numbers of down-regulated genes were comparable between the two lines. There were 59 and 85 genes suppressed in DSY79 and DSY, respectively, by low-P stress (Fig. 4B). We listed these differentially expressed genes with predicted functions in Supplementary Tables S1 and S2, and S3 and classified them into different groups, including phosphate uptake and utilization, metabolism, cell growth and cell wall structure, transcriptional factors, transporters, stress-related genes, and signaling genes, according to their predicted functions. Interestingly, we found that the acid phosphatase gene expressed higher in the low-P tolerant line DSY2 as compared with the other line, which is consistent with the enzyme activity assay (Table S3 and Fig. 3B).

### Root proteomic analysis of contrasting inbred lines

**Figure 5:**
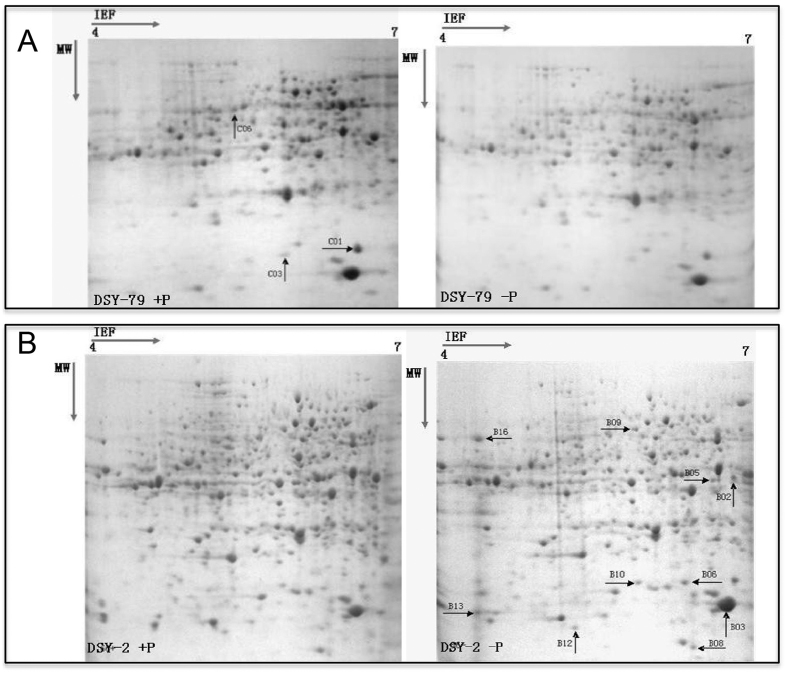
Two-D gel maps of DSY2 and DSY79 roots. (**A**) Two-D gel maps of the low-P sensitive line DSY79 (**A**) and low-P tolerant line DSY2 (**B**) after being treated under normal or low-P conditions.

We used the 2D-Gel based proteomics approach to study differentially accumulated proteins of low-P tolerant and sensitive inbred lines under both normal and low-P conditions to identify the candidate proteins that are responsible for the low-P adaptation between the two lines. The 2D-gel results showed that 8 and 3 proteins were differentially accumulated in DSY2 and DSY79, respectively, upon low-P treatment. We used the mass spectrometry to identify the differentially accumulated proteins (Fig. 5). As shown in Table 1, in the DSY79, the putative cystain, regulator of ribonuclease activity A, and an uncharacterized protein were reduced upon low-P treatment. In DSY-2, eight proteins were induced upon low-P treatment (Table 2). Interestingly, among the 8 induced proteins, there were two ethylene synthesis-related proteins, 1-aminocyclopropane-1-carboxylate oxidase 1 and acc oxidase, induced in low-P treatment in DSY2. The previous study suggests that ethylene, known to be involved in cell expansion and hair root development, has a role in mediating nutrient deficiency response^8^. Our results suggest that DSY2 may confer tolerance to low-P stress partially via modulating ethylene synthesizing.

## Discussions

Due to the importance of P throughout the whole plant life cycle and crop productivity, it is necessary to understand how plants respond to P starvation in order to design more effective breeding programs to produce crop varieties with enhanced Pi uptake and use efficiency. Here we took advantages of the diversity of maize germplasms and analyzed how two contrasting maize inbred lines respond to P-deficiency using the combination of physiological, transcriptomic and proteomic approaches.

Plants have developed elaborate mechanisms to adapt to P-deficiency stress, including a range of morphological, physiological, and biochemical changes enable plants to cope with P starving conditions^3^. Among them, root morphology is a key component in the P-deficiency response strategies. Under P-deficient conditions, plants modify their root development as a consequence of alterations in cell growth and expansion to increase the absorptive surface area. Field trails and greenhouse experiments with maize have shown that certain genotypes that develop more lateral roots and shallow root systems are better adapted to low-P stress^9^. Our results suggest that the low-P stress enhanced the total root length, surface area and volume for both low-P sensitive and resistant inbred lines under hydroponic conditions (Fig. 2). In *Arabidopsis*, plants show a strong reduction of primary root growth when experiencing P deficiency^9^; however, the formation of lateral roots and root hairs that express high levels of P transporters and phosphatases is enhanced by P limitation^10^. In maize, the effects on root systems by low-P stress are more complicate. Some genotypes respond by increasing the number and length of lateral roots, whereas others show the opposite effect^11^. Since the root analysis program analyzed the total root length including both primary and lateral roots, the results indicate that both DSY2 and DSY79 were able to increase the lateral root growth to adapt to the low-P stress. Interestingly, our results also suggest that the root system of DSY2 was always more robust than that of DSY79 under both normal- and low-P conditions, suggesting the larger root system of DSY2 is more dependent on the genetic background rather than the supplement of P. Previous studies have shown that the plants can benefit from large root systems to adapt to the low-P stress. For example, the tomato and rice plants expressing AVP1 display larger root system and more tolerant to low-P stress^12^. Apparently, the DSY2 benefited from its robust root system to adapt to the low-P stress since larger root system leads to higher accessibility to P (Fig. 2).

The regulation of root morphology under P-deficiency conditions is mediated by many factors such as auxin signaling and expansins^2^,^13^. Interestingly, our results showed that the expression level of auxin-responsive genes was significantly higher in DSY2 as compared with DSY79, suggesting modulating auxin-signaling might be one strategy that DSY2 applied to cope with P-starvation. Therefore, further studies may focus on analyzing auxin distribution transport using the maize fluorescence-based reporter lines from the maize cell genomic resources^14^,^15^. Besides the auxin, recent studies also report that the cell wall proteins expansins that regulate cell division and expansion rates may play a critical role in the root architecture responses to low-P stress^11^,^16^. For example, overexpression of a soybean β-expansin *GmEXB2* in *Arabidopsis* increased both root growth and P uptake at both low and high P levels^16^. Our transcriptomic data suggest that the β-expansin gene was induced by the low-P stress in DSY2 but not DSY79, indicating regulation of expansin production might be contributing to the low-P tolerance of DSY2.

Ethylene is another plant hormone has been shown to connect with P-starvation by mediating P nutrient deficiency response^8^. Previous studies in *Arabidopsis* and beans show that ethylene maintains root growth under P-deprivation conditions, indicating the root system acclimates to Pi deficiency by changing the signal transduction pathway connecting ethylene levels to root growth^3^,^17^,^18^. In addition, the production of ethylene in *P. vulgaris* roots grown in P starvation is two-fold higher than that in normal P conditions^3^. Our transcriptomic data suggest that the ethylene-responsive factor-like protein 1 gene was induced by the low-P stress in DSY2 but not DSY79 (Supporting Table S1). Consistently, the proteomics data also showed that the accumulation of two enzymes, 1-aminocyclopropane-1-carboxylate oxidase 1 and acc oxidase, which are critical for ethylene biosynthesis were increased in DSY2 under the low-P condition (Table 2). Therefore, both transcriptomic and proteomic data indicate that the ethylene signaling transduction is more active in DSY2 under low-P condition. The effects of ethylene on root growth are probably mediated through auxin because ethylene stimulates the biosynthesis and transport of auxin^19^. Therefore, there might be sophisticated interactions between the two hormones in DSY2 to modify the root growth and architecture in the response to P starvation. Taken together with the induction of auxin- and ethylene-related genes in DSY2 under the low-P condition, our results suggest that the modulation of ethylene and auxin signaling transduction might be responsible for the low-P tolerance for DSY2.

Alternation of P recycling is another strategy that plants respond to P-starvation besides changing root morphology. One of the largest P pools in plants is stored as phospholipids, which are key components of cell membranes. To provide enough phosphate to maintain the metabolism under P-deficient conditions, one of the biochemical strategies that plants take is an increase of Pi mobilization and recycling activity within the plants, such as hydrolysis of phospholipids^5^. This process requires increased production of acid phosphatases, which is critical for phospholipids hydrolysis. Our transcriptomic results suggest that the acid phosphatase gene was induced in the P-tolerant line, DSY2, but not in the P-sensitive-line DSY79 (Supporting Tables S1 and 2). Consistently, we found that the DSY2 roots showed higher acid phosphate activity, indicating the different phosphate recycling capability between the two lines may partially responsible for their different responses to low-P stress.

The transcriptomic and proteomic studies showed that some development-related signaling genes/proteins that were expressed higher in DSY2 than DSY79 under the low-P condition, such as heterotrimeric G proteins and CLAVATA1 (Supporting Table S3). For instance, the heterotrimeric G-protein α and β-like subunits were 6.4 and 3.1 times higher, respectively, in DSY2 than DSY79 under low-P condition (Supporting Table S3). The heterotrimeric G-protein complex, consisting of Gα, Gβ and Gγ subunits, is a molecular mechanism, which transmits signals from transmembrane receptors to downstream target proteins^20^,^21^. It plays roles in many aspects of plant development, hormone-sensing and nutrient uptake^21^,^22^. The recent study has identified a G protein γ subunit of rice DEP1 regulates nitrogen utilize efficiency^22^. Thus, it will be interesting to investigate if G proteins contribute to P-utilize efficiency. The CLAVATA signaling pathway is essential for the regulation of meristem activities in plants^23^,^24^,^25^,^26^. The CLAVATA pathway consists of CLE peptides which interacting with Leucine-rich repeats receptor-like kinases (LRR-RLKs) such as CLVATA1 and other co-receptors^27^. Recent studies show that CLE-CLAVATA1 peptide-receptor signaling module regulates the expansion of plant root systems in a nitrogen-dependent manner^27^,^28^,^29^. Our results indicate that the CLAVATA-CLE signaling may also be involved in P starvation responses. The CLAVATA proteins regulate meristem development partially via the heterotrimeric G protein γ subunit^20^, and the heterotrimeric G protein γ subunit has been reported to mediate nitrogen utilization^22^. Taken together of the information, it will be interesting to understand how are the CLVATA and G protein signaling responsible for the low-P tolerance of DSY2 inbred line.

## Conclusions

We demonstrate here that the two contrasting inbred lines responded to P-starvation differently from their morphology, physiology, gene expression, and protein accumulation aspects. The genes identified from the study might be used for further functional characterization to understand the mechanisms underlying P utilize. In addition, they may also be used by the maize geneticists or breeders to develop genetic markers that associate with P-utilize efficiency. Overall, the new insights generated in this study will be useful toward the improvement of P utilize efficiency in maize.

## Materials and Methods

### Screening the low-P tolerance maize inbred lines in field

One hundred and sixteen maize inbred lines were collected from northeast, Huabei, northwest, and southwest of China (Supporting Table S4). The field trial was conducted at the Langfang Experimental Station of Chinese Academy of Agricultural Sciences (Coordinate: E116.35′, N39.36′). The seeds were sowed on April 27^th^, 2007 and the plants were harvested on September 10^th^, 2007. The soil used for the experiment contains relatively low P with total P content as 0.69 g/kg and the available P content as 2.77 mg/kg. We used random block experimental design with 3 replicates and 2 levels (normal P and low P) for each inbred line. We applied 120 kg ha^−1^ P_2_O_5_ as normal P treatment, whereas no P_2_O_5_ was applied for the low-P treatment. The 100-grain weight was determined for each inbred line under normal or low-P treatment.

### Maize hydroponic culture

Two maize inbred lines displayed extreme low-P sensitive (DSY-79) or low-P tolerance (DSY-2) were selected for further experiments. The hydroponic experiment was conducted in the greenhouse of Chinese Academy of Agricultural Sciences with the light cycle of 16 h light and 8 h dark. The temperature was maintained between 26–30 °C. The maize seeds were sowed in moisture sands in May 20^th^, 2010 then transplanted to the modified Hoagland’s hydroponic solution with or without P at 3-leaf stage. The pH was adjusted daily to 6.5 using 1 M HCl. The solution was replaced every three days. The plants were harvested at the different time points as indicated in different experiments. The concentration for normal-P treatment was CaCl_2_ 5 mM, NH_4_Cl 15 mM, KH_2_PO_4_ 1 mM, MgSO_4_·7H_2_O 2 mM, KCl 5 mM, H_3_BO_3_ 46 μM, MnCl_2_ 9 μM, ZnSO_4_·7H_2_O 0.7 μM, CuSO_4_·5H_2_O 0.2 μM, H_2_MoO_4_.H_2_O 0.05 μM, and 0.1 μM Fe-EDTA. For the low-P treatment, the KH_2_PO_4_ was replaced by 1 mM KCl. All the experiments described below used the plants materials of hydroponic culture and had 3 biological replicates.

### Root morphology

The root morphology was acquired by using EPSON Perfection 4990 scanner (EPSON, US) 10 days after hydroponic culturing. The root length, surface area and volume were analyzed using the digital image analysis system “WinRHIZO Pro v4.0 (Regent Instruments Inc., Canada)”.

### Root vitality and acid phosphatase activity

The root vitality was determined using triphenyltetrazolium chloride (TTC) method with slightly modification^30^. One hundred 1 cm-root tips were collected and incubated in the solution contain 5 ml 0.4% TTC and 0.1 M PBS (pH 7.0) at 37 °C for 1 h. Then 20 ml methanol was added followed by incubating at 37 °C for 6 h. The total extracted solution was measured with a spectrophotometer at 485 nm.

The acid phosphatase activity assay was conducted according to Yun and Kaeppler with some modifications^31^. Briefly, the root of DSY79 and DSY2 were harvested 8 days after hydroponic treatment and ground in liquid nitrogen followed by adding extraction buffer contains 0.1 M sodium acetate (NaOAc), 0.1 mM Phenylmethylsulfonyl fluoride (PMSF), 5 mM DL-Dithiothreitol (DTT), 5 mM sodium chloride (NaCl), pH 5.2. 1/10 ml of protein extraction was added to 2.9 reaction buffer contains (1.5 mM NaOAc, 1.5 mM p-nitrophenol phosphate (p-NPP), 5 mM NaCl, pH5.2) and incubated at 37 °C for 30 min. The reaction was quenched by adding 1 ml 1 M NaOH, followed by centrifuging at 4 °C for 10 min. The OD of the supernatant was measured by spectrophotometer at 400 nm wavelength.

### RNA extraction and microarray

Total RNA was extracted from root tissue of normal-P or low-P treated DSY79 and DSY9 maize plants using Trizol (Invitrogen) according to the manufacturer’s instruction. The RNA quality of these samples was assessed by Agilent Bioanalyzer 2100 (Agilent Technologies). Total RNA was processed for the microarray hybridization using the Affymetrix GeneChip 3′ IVT Express Kit (Affymetrix). The resultant biotinylated copy RNA was fragmented and hybridized to the GeneChip Maize Genome Arrays (Affymetrix). The arrays were washed, stained, and scanned. The data were imported into GeneSpring software (Agilent Technologies) and normalized with the 50% percentile shift. The unpaired *t*-test was used to calculated *P* values. Genes with *P* < 0.05 and 2-fold or greater change in expression were considered to be differentially expressed and gathered for further analysis. Annotations of differentially expressed genes were obtained by BLAST search comparison with the National Center for Biotechnology Information (NCBI).

### Protein extraction and 2-D electrophoresis

The root protein extraction and 2-D electrophoresis procedures were described by Nazir^32^. Briefly, 2–3 g root tissues were grinded in liquid nitrogen and total proteins were extracted in 10 ml protein extraction buffer containing 100 mM Tris-HCl (pH 8.0), 5 mM EDTA (pH 8.0), 1 mM PMSF and 2% beta-mercaptoethanol (β-ME), followed by centrifuging at 10,000 g for 15 min. The protein extracts were precipitated by adding 4-volum 10% ice-cold TCA followed by incubating at −20 °C for 4 h. Then the samples were centrifuged at 10, 000 g 4 °C for 15 min. The pellets were then washed with acetone and dissolved in Laemmli sample buffer for 2D-Gel analysis according to Zhang *et al*.^6^. The gels were stained with coomassie brilliant blue and the scanned images were analyzed using PDQuest software (Bio-Rad).

### Mass spectrometric analysis

The differential expressed protein spots were collected and digested with trypsin followed by the MALDI-TOF mass spectrometry analysis as described by Nazir *et al*.^32^.

### Statistical Analyses

Three biological replicates were applied for all experiments that were described above. A two-tailed Student’s *t*-test using 95% significance was performed for statistical analyses.

## Additional Information

**How to cite this article**: Jiang, H. *et al*. Revealing new insights into different phosphorus-starving responses between two maize (*Zea mays*) inbred lines by transcriptomic and proteomic studies. *Sci. Rep.* **7**, 44294; doi: 10.1038/srep44294 (2017).

**Publisher's note:** Springer Nature remains neutral with regard to jurisdictional claims in published maps and institutional affiliations.

## Supplementary Material

Supplementary Information

## Figures and Tables

**Table 1:** The predicted functions of proteins suppressed by low-P stress in DSY79.

Spot No.	Protein Name	Score	GI No.	MW(Da)	pI
C01	putative cystatin	99	gi|71794639	24548	6.14
C03	regulator of ribonuclease activity A	116	gi|226502502	17986	5.78
C06	uncharacterized protein	176	gi|226510596	52991	6.00

**Table 2:** The predicted functions of proteins induced by low-P stress in DSY2.

Spot No.	Protein Name	Score	GI No.	MW(Da)	pI
B02	1-aminocyclopropane-1-carboxylate oxidase 1	172	gi|226494732	34473	4.99
B05	acc oxidase	228	gi|38607363	35076	5.40
B06	eukaryotic translation initiation factor 5A	210	gi|162458009	17366	5.61
B08	calmodulin	164	gi|226530441	24449	4.40
B09	S-adenosyl-l-homocysteine hydrolase	184	gi|226491362	53117	5.63
B10	kinesin family protein	89	gi|225556906	199317	5.34
B16	isopentenyl transferase IPT8	174	gi|166033754	40632	9.17
B03	unknown	384	gi|223973509	23687	11.77
B12	unknown	112	gi|226505300	17192	4.85
B13	unknown	235	gi|219887679	12103	9.20
