# Supplementary material for: Revealing new insights into different phosphorus-starving responses between two maize (Zea mays) inbred lines by transcriptomic and proteomic studies
Source: Sci Rep. 2017 Mar 9;7:44294. doi: 10.1038/srep44294 (PMC5343578; doi:10.1038/srep44294)
Supplement: Supplementary Information [file srep44294-s1.pdf]

Supplementary Information

Revealing new insights into different phosphorus-starving responses between two maize (*Zea mays*) inbred lines by transcriptomic and proteomic studies

Huimin Jiang[1#], Jianfeng Zhang[1#], Zhuo Han[2#], Juncheng Yang[1], Cailin Ge[2*], Qingyu Wu[1,3,*]

[1] *Institute of Agricultural Resources and Regional Planning, the Chinese Academy of Agricultural Sciences, Beijing 100081, People's Republic of China*

[2] *College of Bioscience and Biotechnology, Yangzhou University, Yangzhou 225009, People's Republic of China*

[3] *Current address: Cold Spring Harbor Laboratory, 1 Bungtown Road, Cold Spring Harbor, New York, United States*

[#] *The first three authors contributed equally to this work.*

[*] **To whom correspondence should be addressed to:**

Qingyu Wu

Tel.: 86 (0)10 82106203

Email: qwu@cshl.edu

Or Cailin Ge

Tel.: +86 (0)514 87979365

Email: gecailin10@163.com

Table S1 The genes that were induced by low-P stress in DSY2 (Low-P tolerant) with known functions.

Category	Gene	Coding protein	Folds increased
Phosphate uptake and utilization	LOC100281846	phospholipase A2	2.025
	LOC100280563	acid phosphatase	2.860
	LOC100280644	ribose-phosphate pyrophosphokinase 4	2.107
	LOC100282754	GMP synthase	4.110
	LOC100285534	ATP binding protein	2.354
	LOC542713	ferredoxin-NADP reductase precursor	2.066
	hsp1	heat shock protein1	2.318
Metabolism	Plant hormone metabolism		
	LOCOPR1	12-oxo-phytodienoic acid reductase	2.023
	LOCOPR3	12-oxo-phytodienoic acid reductase	3.705
			3.013
	LOC100283015	methylthioribose kinase	2.793
	IDP155	IDP155 protein	2.503
	LOC100281313	abscisic stress ripening protein 2	2.368
	LOC100285870	auxin-repressed 12.5 kDa protein	4，047
	Secondary metabolism		
	LOC100283365	3-N-debenzoyl-2-deoxytaxol N-benzoyltransferase	2.180
	LOC100281303	limonoid UDP-glucosyltransferase	2.000
	irl1	isoflavone reductase-like1	2.271
	TPS8	Terpene synthase 8	6.988
	Other metabolisms		
	LOC100282568	adenine phosphoribosyltransferase 1	2.149
	LOC100283128	lipid binding protein	2.527
	LOC100281735	uridylate kinase	2.796
	gln1	glutamine synthetase1	2.627
	LOC100284999	myristoyl-acyl carrier protein thioesterase	2.046
	LOC100282341	hydrolase	2.345
	LOC100282518	actin-depolymerizing factor	4.717
	LOC100282060	actin-depolymerizing factor	4.831
	LOC100285831	cortical cell-delineating protein	2.011
	cesa10	cellulose synthase10	2.054
	cesa11	cellulose synthase 11	2.539
Cell growth and cell wall structure	expB7	beta-expansin 7	2.490
	LOC100283877	glycine-rich protein A3	2.245
	LOC100280652	verprolin protein	13.459
	LOC100285460	homeobox-leucine zipper protein ATHB-6	2.001
	LOC100286113	germin-like protein subfamily 1 member 11	5.301
	LOC100280983	CCCH transcription factor	3.078
	LOC100283357	AP2 domain containing protein	2.178
	LOC100281239	dehydration-responsive element-binding protein 1D	2.835

	lg3	liguleless3	2.059
	LOC100285453	GATA transcription factor 22	2.416
Transcription factor	LOC100280523	ethylene-responsive factor-like protein 1	2.284
	DMAS1	deoxymugineic acid synthase1	3.252
	ys1	yellow stripe	2.963
	NAS1	NAS1 protein	7.299
	NAS1	NAS1 protein	7.107
	LOC100286321	copper transporter 1	4.955
Transporter	LOC100281339	zinc transporter 4	2.134
	LOC100285636	carbohydrate transporter/ sugar porter	2.069
	LOC542332	permease 1	6.477
	LOC100280888	aminomethyltransferase	2.061
	LOC100281369	glutathione S-transferase	2.128
	gst19	glutathione transferase19	2.308
	gst31	glutathione transferase31	2.249
	LOC100282094	L-ascorbate oxidase	4.130
	Hb2	hemoglobin 2	2.200
Stress-related genes	LOC541815	hemoglobin	5.900
	mtl2	metallothionein2	2.310
	LOC100282059	metallothionein-like protein type 2	2.501
	LOC732827	Wound responsive protein-like	2.823
	LOC100286345	low-molecular-weig-ht cysteine-rich protein	3.758
	LOC100284348	Bowman-Birk type bran trypsin inhibitor	3.428
	LOC100280692	aldehyde dehydrogenase 3B1	21.142
	LOC100285647	blue copper protein	2.883
	LOC100282634	rhodanese-like domain containing protein	5.817
	LOC100285847	lysine decarboxylase-like protein	2.291
	LOC100282094	L-ascorbate oxidase	2.056
	LOC100281444	protein kinase domain containing protein	3.959
	LOC100285871	systemin receptor SR160	2.003
	rop5	Rho-related protein from plants 5	2.108
	LOC100285461	AP-1 complex subunit sigma-2	3.823
	LOC100282368	CREG1 protein	2.317
Cell signaling	LOC100284388	calmodulin binding protein	2.146
	LOC100285871	systemin receptor SR160	2.003
	rop5	Rho-related protein from plants 5	2.108
	LOC100285461	AP-1 complex subunit sigma-2	3.823
	LOC100282368	CREG1 protein	2.317
	LOC100284388	calmodulin binding protein	2.146

Table S2 The genes that were induced by low-P stress in DSY79 (Low-P sensitive) with known functions.

Category	Gene	Coding protein	Folds increased
Phosphate uptake and utilization	LOC732717	inorganic phosphate transporter 3	2.449
Metabolism	LOC100282866	cellular retinaldehyde-binding/triple function, C-terminal	2.187
	AsnS4	Asparagine synthetase 4	2.187
	LOC100282556	anthocyanidin 3-O-glucosyltransferase	2.089
	LOC100282766	aldo-keto reductase/ oxidoreductase	2.398
Cell growth and cell wall structure	xet1	xyloglucan endotransglycosylase homolog1	2.324
Transporter	LOC100286321	copper transporter 1	3.719
Stress-related genes	Hb2	hemoglobin 2	2.687
	LOC100284675	peroxidase 1	2.389
	LOC100281825	basic endochitinase C	2.219
	LOC100283929	jasmonate-induced protein	2.024

Table S3 The genes that were up-regulated in DSY-2 (low-P tolerant) as compared with DSY-79 (low-P sensitive) under low-P conditions.

Category	Genes	Coding proteins	Folds increased
Phosphate uptake and utilization	LOC100281920	regulator of ribonuclease activity A	4.385
	LOC100280563	acid phosphatase	2.846
	abp1	auxin binding protein1	2.816
	LOC100283062	phi-1-like phosphate-induced protein	2.705
	LOC100281365	mannose-6-phosphate isomerase	2.547
	aox3	alternative oxidase AOX3 precursor	2.434
	LOC100285552	ethanolaminephosphotransferase	2.415
	LOC100281325	bundle sheath defective2	13.576
	mas1	malate synthase1	6.292
	LOC100282754	GMP synthase	3.880
	LOC100285737	thiamin pyrophosphokinase 1	3.723
	LOC100280499	2-isopropylmalate synthase B	3.400
	LOC100281248	chlorophyll a-b binding protein 2	2.879
	LOC100280485	RNA binding protein	15.963
	LOC100282349	LSM7-like	7.443
	LOC100284093	cytosolic 5-nucleotidase III	5.438
	pdc1	pyruvate decarboxylase1	2.669
Metabolism	Protein and amino acids metabolisms		
	rpp2a-3	acidic ribosomal protein P2a-3	116.95
	LOC100280637	polyubiquitin containing 7 ubiquitin monomers	60.932
	LOC100281802	60S ribosomal protein L35	49.022
	LOC100280556	60S ribosomal protein L27	3.068
	rps28	Ribosomal protein S28	3.184
	LOC100280657	60S ribosomal protein L4	2.050
	LOC542088	nucellin-like aspartic protease	6.588
	LOC100281059	glutamyl-tRNA synthetase, cytoplasmic	6.383
			2.630
	hsp18a	18 kda heat shock protein18a	2.849
	LOC100281292	ubiquitin-protein ligase	2.280
	EF1-A	Elongation factor 1-alpha	2.720
	LOC100280633	aspartic proteinase nepenthesin-2	2.514
	LOC100284155	nuclear protein	2.871
	LOC100281935	negatively light-regulated protein	2.367
	LOC100283647	non-imprinted in Prader-Willi/Angelman syndrome region protein 1	2.297
	LOC100286211	mitochondrial glycoprotein	2.048
	Plant hormone metabolisms		
	LOC100284018	IDP158 protein	4.292
	IDP2449	IDP2449 protein	2.206
	IDP155	IDP155 protein	2.112
	LOC100284720	gibberellin-regulated protein 1	3.965
	LOC100286041	gibberellin-regulated protein 2	2.253
	LOC100286190	auxin-independent growth promoter-like protein	2.603
	LOC100285745	auxin efflux carrier component 1c	2.023
	Secondary metabolisms		
	LOC100282888	S-adenosylmethionine decarboxylase	3.023

		proenzyme	
	LOC100280589	agmatine coumaroyltransferase	64.321
	LOC100280459		8.818
	LOC100283528	agmatine deiminase	2.056
	TPS8	Terpene synthase 8	17.813
	dxs2	deoxy xylulose synthase 2	4.858
	LOC100282968	phosphomevalonate kinase	3.015
	OPR3	12-oxo-phytodienoic acid reductase	12.681
	LOC100284721	3-N-debenzoyl-2-deoxytaxol N-benzoyltransferase	7.691
	a2	anthocyaninless2	4.745
	irl1	isoflavone reductase-like1	4.314
	c2	chalcone synthase	3.747
	LOC100286107	dihydroflavonol-4-reductase	3.481
	LOC100284846		2.168
	LOC100280732	4,5-DOPA dioxygenase extradiol	2.074
	LOC100284999	myristoyl-acyl carrier protein thioesterase	2.064
	Other metabolisms		
	LOC100284997	lipid binding protein	4.397
	LOC100282744	omega-6 fatty acid desaturase, endoplasmic reticulum isozyme 2	3.278
	thi1-2	thiamine biosynthetic enzyme	2.049
	LOC100285474	single-stranded DNA-binding protein	2.608
	LOC100285956	structural constituent of ribosome	2.097
	LOC100285105	hydrolase	2.235
Cell growth and cell wall structure	LOC100283492	fiber protein Fb2	2.420
	LOC542725	Glycine-rich protein	2.012
	LOC100286345	low-molecular-weight cysteine-rich protein LCR70	2.791
	LOC100284638	PIF-like orf1	73.951
	expa2	alpha expansin2	4.636
	LOC100285844	expansin-like 3	2.954
	cesa10	cellulose synthase10	3.409
	cesa11	cellulose synthase 11	2.996
	cesa12	cellulose synthase catalytic subunit 12	3.105
	LOC100283442	glucan endo-1,3-beta-glucosidase A6	24.645
	LOC100280947	cytidine/deoxycytidylate deaminase family protein	4.397
	tub6	Beta-6 tubulin	2.642
	LOC542424	gamma-tubulin	6.884
	LOC541616	root cap-specific glycine-rich protein	6.125
	GRP5	glycine-rich protein GRP5	3.926
	LOC100282701	anther-specific proline-rich protein APG	5.212
	LOC100282281	endo-1,3;1,4-beta-D-glucanase	2.077
	czog1	cis-zeatin O-glucosyltransferase1	3.106
	xet1	xyloglucan endotransglycosylase homolog1	3.130
	LOC100284565	stem 28 kDa glycoprotein	2.225
	LOC100284989	fasciclin-like arabinogalactan protein 10	2.214
	LOC100283617	dihydrolipoyllysine-residue acetyltransferase component of pyruvatedehydrogenase complex	2.016

	H4C14	Histone H4	2.075
Transcription factor	LOC100280600	IAA24-auxin-responsive Aux/IAA family member	6.431
	LOC100285285	transposon protein	5.365
	LOC100283216	retrotransposon protein SINE subclass	4.881
	LOC542428	Dof2	4.992
	LOC100280983	CCCH transcription factor	4.286
	CAF1	CRS2-associated factor 1	4.217
	maf1	MFP1 attachment factor 1	4.089
	LOC100283082	EF hand family protein	3.178
	GRF9	Growth-regulating factor 9-like	2.598
	ham101	histone acetyl transferase MYST family 101	2.493
	LOC100281239	dehydration-responsive element-binding protein 1D	2.042
	OBF3.1	ocs-element binding factor 3.1	2.012
	LOC542486	R2R3MYB-domain protein	2.022
Transporter	nip2b	NOD26-like membrane intrinsic protein2	25.600
	LOC100282864	copper chaperone	10.587
	LOC100283229	copper ion binding protein	6.142
	LOC541917	sulfate transporter ST1	2.747
	LOC542332	permease 1	6.873
	LOC100281381	S-adenosylmethionine-dependent methyltransferase	5.694
	LOC100285636	carbohydrate transporte	3.910
	LOC100285394	sugar transport protein 5	2.629
	LOC100282577	plant integral membrane protein TIGR01569 containing protein	3.808
	LOC100284275	VAMP protein SEC22	3.376
	LOC100282484	mitochondrial inner membrane protease subunit 1	2.056
Stress-related genes	LOC100282529	disease resistance response protein 206	57.910
	LOC100283996	protein induced upon tuberization	32.495
	LOC100281212	cysteine-type peptidase	2.511
	LOC100280692	aldehyde dehydrogenase 3B1	27.752
	gst10	glutathione transferase10	23.624
	sod9	Superoxide dismutase9	5.052
	LOC100282059	metallothionein-like protein type 2	4.872
			4.308
	LOC541847	glutathione S-transferase GST 38	4.371
	LOC541843	glutathione S-transferase GST 32	4.260
	LOC542630	glutathione S-transferase GST 15	2.337
	gst30	glutathione transferase30	2.519
	LOC100285908	peroxisomal-coenzyme A synthetase	3.017
			2.126
	LOC100136885	chloroplast Cu-Zn superoxide dismutase	2.401
	Umi3	sesquiterpene cyclase-like	11.810
	inra2(prp)	defence-related protein	11.620
	LOC541949	Betaine aldehyde dehydrogenase	11.104
	LOC100283057	bax inhibitor 1	16.232
	LOC100284518	seed maturation protein	2.774

	LOC100283251	seed specific protein Bn15D17A	2.399
	LOC100281945	Bowman-Birk type wound-induced proteinase inhibitor WIP1	5.613
	LOC100284348	Bowman-Birk type bran trypsin inhibitor	3.072
	rf2d	cytosolic aldehyde dehydrogenase RF2D	3.953
	MRP1	Multidrug resistance associated protein 1	3.921
	LOC100282066	wound induced protein	3.244
	LOC100285556		3.003
	LOC100282583	erwinia induced protein 2	2.739
	per-1	1-Cys peroxiredoxin antioxidant	2.965
	LOC100283195	disease resistance response protein 206	2.586
	LOC541688	trypsin inhibitor	2.681
	hsbp1	herbicide safener binding protein1	2.619
			2.328
	LOC100282796	physical impedance induced protein	2.146
Signaling	LOC542644	plasma membrane integral protein ZmPIP2-2	123.45
	LOC542647	tonoplast membrane integral protein ZmTIP4-4	2.567
	LOC100280841	seven transmembrane domain protein	34.756
	LOC100283222	chemocyanin	25.419
	LOC100284336		2.966
	LOC100284295	ADP-ribosylation factor	10.030
	LOC100284718	guanine nucleotide-binding protein alpha-1 subunit	6.449
	LOC100281713	guanine nucleotide-binding protein beta subunit-like protein 1	3.141
	LOC100285335	ABA induced plasma membrane protein PM 19	3.001
	LOC100280966	BRASSINOSTEROID INSENSITIVE 1-associated receptor kinase 1	2.870
	LOC100281253	receptor protein kinase CLAVATA1	2.829
	LOC100284388	calmodulin binding protein	2.565
	LOC542227	Calcium-dependent protein kinase	2.392
	crt2	Calreticulin2	2.087
	LOC100280762	cyclin-dependent kinase inhibitor 2	2.524
	LOC100280945	B12D protein	2.451
	LOC100283680	gibberellin receptor GID1L2	2.367
	LOC100281786	protein kinase	2.087
	ptk1	protein kinase1	2.026
	LOC100285495	CBL-interacting serine/threoni ne-protein kinase 15	2.148

Table S4 Names of the inbred lines that were used for the filed trial.

Public Names	Internal Numbers
CAL158	DSY01
Ye107	DSY02
Lu2548	DSY03
DHuang212	DSY04
515	DSY05
JI853	DSY06
DanHuang02	DSY07
MoQun14	DSY08
Yu12	DSY09
ZhongHuang204	DSY10
B73	DSY11
DuoHuang29	DSY12
Zhong451	DSY13
CN962	DSY14
CN165	DSY15
ZaC546	DSY16
Dong237	DSY17
E28	DSY18
Dong46	DSY19
C86052	DSY20
Shen5003	DSY21
Shen118	DSY22
Ji53	DSY23
Hai921	DSY24
LuYuan133	DSY25
K22	DSY26
4379	DSY28
Zhong106	DSY30
32	DSY31
ZhongHuang68	DSY32
501	DSY33
CN7379	DSY34
CA339	DSY35
Chuan2191	DSY36
Chuan273	DSY37
Chuan321	DSY38
Qi319	DSY39
Ji412	DSY40
Ji419	DSY41

Ji465	DSY42
Ji4112	DSY43
Liao3053	DSY44
LiaoBai371	DSY45
Shen136	DSY46
Dan598	DSY47
Dan599	DSY48
Dong156	DSY49
Dong91	DSY50
Hai014	DSY51
Lian87	DSY52
Q1261	DSY53
3189	DSY54
196	DSY55
Y8G	DSY56
835	DSY57
CA375	DSY58
CA344	DSY59
CD12	DSY60
CD13	DSY61
CD14	DSY62
CAL99	DSY63
CAL70	DSY64
CAL73	DSY65
Ji846	DSY66
CA112	DSY67
374	DSY68
Guan17	DSY69
H30	DSY70
H66	DSY71
Zhi41	DSY72
910412	DSY73
2548	DSY74
EL314	DSY75
EL318	DSY76
EL321	DSY77
CML476	DSY78
CML483	DSY79
CML484	DSY80
CML487	DSY81
CML489	DSY82
CML490	DSY83
CML491	DSY84

CML493	DSY85
CML495	DSY86
CML496	DSY87
CML154	DSY97
CML163	DSY98
CML194	DSY99
CML91	DSY100
Zheng58	DSY101
Wu314	DSY102
